# Microbial Removal of the Pharmaceutical Compounds Ibuprofen and Diclofenac from Wastewater

**DOI:** 10.1155/2013/325806

**Published:** 2013-11-19

**Authors:** Alette Langenhoff, Nadia Inderfurth, Teun Veuskens, Gosse Schraa, Marco Blokland, Katarzyna Kujawa-Roeleveld, Huub Rijnaarts

**Affiliations:** ^1^Department of Environmental Technology, Wageningen University, P.O. Box 17, 6700 EV Wageningen, The Netherlands; ^2^Laboratory of Microbiology, Wageningen University, Dreijenplein 10, 6703 HB Wageningen, The Netherlands; ^3^RIKILT, Wageningen UR, Akkermaalsbos 2, 6708 WB Wageningen, The Netherlands

## Abstract

Studies on the occurrence of pharmaceuticals show that the widely used pharmaceuticals ibuprofen and diclofenac are present in relevant concentrations in the environment. A pilot plant treating hospital wastewater with relevant concentrations of these pharmaceuticals was evaluated for its performance to reduce the concentration of the pharmaceuticals. Ibuprofen was completely removed, whereas diclofenac yielded a residual concentration, showing the necessity of posttreatment to remove diclofenac, for example, activated carbon. Successively, detailed laboratory experiments with activated sludge from the same wastewater treatment plant showed bioremediation potential in the treatment plant. The biological degradation pathway was studied and showed a mineralisation of ibuprofen and degradation of diclofenac. The present microbes were further studied in laboratory experiments, and DGGE analyses showed the enrichment and isolation of highly purified cultures that degraded either ibuprofen or diclofenac. This research illuminates the importance of the involved bacteria for the effectiveness of the removal of pharmaceuticals in a wastewater treatment plant. A complete removal of pharmaceuticals from wastewater will stimulate water reuse, addressing the worldwide increasing demand for clean and safe fresh water.

## 1. Introduction

Since the last 10–20 years the quality of surface water systems and their interacting groundwater systems is increasingly under pressure as a result of new groups of chemicals entering these natural water systems. The occurrence of organic micropollutants such as pharmaceuticals, synthetic hormones, and personal care products has the increasing attention of drinking water companies and water resource institutions. Furthermore, the development of analytical techniques to measure these compounds at low concentrations has accelerated this awareness. In the last 40 years, technologies for the removal and recovery of bulk substances from wastewater effluents, such as organics and nutrients like phosphorus and nitrogen, have been developed and implemented into the water cycle infrastructure. As these technologies are designed to deal with bulk load emissions, many organic micropollutants are not removed during the passage through these systems [1]. 

Organic micropollutants such as pharmaceuticals enter the water cycle either as the original compound or in a metabolized form at low loads [2], resulting in relatively low concentrations, that is, in the ng/L–*μ*g/L range [3, 4]. The upper range of these concentrations is found in wastewater that originates from the so-called hot-spots such as hospitals or elderly houses. These concentrations have most likely been present in the water since many years, and their levels have only recently been quantified and acknowledged as a potential ecological risk. The recent development of new analytical techniques has allowed us to detect such low concentrations in the environment. Although they are often present in low concentration, various studies into effects on quality and ecological functioning of water systems show that these chemicals form a potential new problem. Their estrogenic and carcinogenic toxicity will impact the quality of ecological life and possibly also of human life [5]. There are currently no legally regulated maximum permitted concentrations of pharmaceuticals in the environment. If we are able to remove these organic micropollutants in a cost effective manner from wastewater, This will reduce their environmental impact on natural water sources and soil. In addition, an optimised water reuse helps to limit the use of fresh water, which is a growing concern in rivers worldwide. 

**Figure 1:**
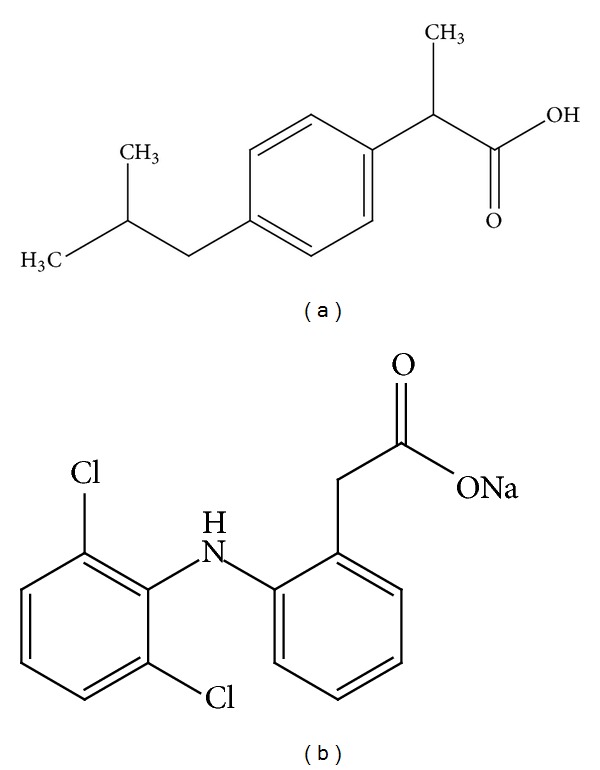
Chemical structure of ibuprofen (a) and diclofenac (b).

Studies on the occurrence of pharmaceuticals show that the widely used pharmaceuticals ibuprofen and diclofenac (Figures 1(a) and 1(b)) are present in relevant concentrations in the environment [4]. Both ibuprofen and diclofenac are non-steroidal drugs, used against pain, fever and inflammation and can be used without prescription. The yearly use of ibuprofen and diclofenac in the Netherlands in 2009 has been estimated at 22.600 kg and 5.200 kg, respectively [6]. 

Efficient removal of low concentrations of ibuprofen and diclofenac from wastewater has to occur at a time scale of minutes to days due to the short retention time in a treatment plant. Currently, highly selective and rapid reactions turned out to be efficient to remove micropollutants, such as advanced oxidation processes (AOPs) that use combinations of reactive oxidants including ozonation, photocatalyses, and ultrasound oxidation [7–10] or adsorbents such as activated carbon [11]. AOPs are characterized by the production of extremely reactive and unselective species such as hydroxyl radicals, which are able to degrade recalcitrant molecules into possible biodegradable intermediate compounds or completely mineralize them into CO_2_, H_2_O, and inorganic ions. However, this may also lead to the formation of potentially harmful by-products, and such oxidation technologies are characterized by a large ecological footprint due to a high energy use [4]. In addition, many of these systems are under research and have yet to be applied on a large scale since there is a lack of good quality data on the mechanisms involved, the influence of operational variables, the reaction kinetics, and reactor design issues. 

In principle, biological techniques can be more robust and cost-effective for the removal of micropollutants compared to oxidation technologies [12], but many micropollutants are not sufficiently removed in the currently operated high organic loaded biological water treatment systems that focus on chemical oxygen demand (COD) and nutrient removal [13].

In the literature, various removal rates are described for the biological removal of both ibuprofen and diclofenac; for example, diclofenac showed low removal rates (21.8 ± 28.5%) in a pilot-scale membrane bioreactor, whereas ibuprofen showed a removal of 99.1 ± 1.8% [14]. In general, ibuprofen reported removal rates are among the highest ones of all pharmaceuticals, as ibuprofen is known for its easy biodegradability, and removal rates of over 95% are often mentioned in lab-scale and wastewater treatment plants [15]. Interestingly, in many cases pharmaceutical loads increased during the wastewater treatment, resulting in a removal efficiency above 100%, due to fluctuating sorption and desorption of the pharmaceuticals to organic matter. Recently, also constructed wetlands were found to effectively remove pharmaceuticals, with ibuprofen removal following predominantly microbial aerobic degradation [16].

More detailed biodegradation studies with batch tests and flow-through soil columns under unsaturated, aerobic conditions also demonstrated biodegradation for pharmaceuticals, such as ibuprofen and diclofenac [17]. Only one study describes anaerobic lab-scale experiments that show 30–60% removal of ibuprofen under anoxic conditions and up to 80% degradation of diclofenac [18]. Recently, the complete aerobic removal of ibuprofen and diclofenac was demonstrated with the white rot fungus *Phanerochaete chrysosporium* in fed-batch bioreactors [19]. This shows that there are currently various bioreactors being tested for their removal capacity which are effective for the treatment of organic micropollutants. 

So far, little is known about bacteria that degrade these pharmaceuticals and the involved biodegradation pathways, for example, only one bacterial strain has been described that degrades ibuprofen and uses ibuprofen as carbon and energy source [20]. Diclofenac has been shown to be biodegradable, but the responsible bacteria are unknown. In addition, the white rot fungus *Phanerochaete chrysosporium* is able to completely degrade ibuprofen and diclofenac [19]. 

The objectives of this study were (1) to determine the removal of ibuprofen and diclofenac in a pilot membrane bioreactor (MBR), (2) to investigate the biological transformation of the pharmaceuticals and the effect of electron acceptor and the presence of an easily degradable substrate (acetate or toluene) on the removal of the pharmaceuticals, and (3) to get more insight in the degradation pathway and the involved bacteria. This allows us to get insight into the biodegradation processes, including enrichment of the responsible bacteria, elucidating the degradation pathways, and evaluation of the removal of these pharmaceuticals in a wastewater treatment plant.

## 2. Materials and Methods

### 2.1. Wastewater Treatment System

Wastewater from a hospital in The Netherlands was treated in a pilot wastewater treatment system. Pretreatment consisted of screening (6 mm) and sieving (0.5 mm wide openings) of the wastewater. The wastewater treatment plant consisted of a membrane bioreactor (MBR), with various posttreatment steps. The bioreactor was aerated with compressed air generated by two compressors and distributed by fine bubble disc aerators. The membrane unit was positioned outside the reactor. The flow of the liquid was outward through the tubular membranes. Nine Hyperflux tubular modules (MO 83G_I8LE BA, Berghof) made of fiber reinforced polymer resin with a membrane area surface of 27.2 m^2^/module were used. The bioreactor was operated at an average temperature of 17.4°C, an organic loading rate of 0.032 kg BOD/kg TS/d, and an HRT of minimal 28 hrs. 

Based on the expected daily amount of wastewater, an average flow rate of 10 m^3^/h was chosen, with a maximum flow of 25 m^3^/h. The total volume of the MBR was 280 m^3^. The plant was designed to treat chemical oxygen demand concentrations of 490–690 mg/L COD and an average concentration of total solids (TS) of 6 g/L, making it an ultra-low loaded reactor. As a result, sludge was discharged in low quantities, resulting in a high sludge retention time (SRT). 

Various posttreatment lines were installed in parallel: (i) granular activated carbon, (ii) ozone treatment with and without H_2_O_2_ addition, (iii) ozone treatment with and without H_2_O_2_ addition, followed by GAC, (iv) UV/H_2_O_2_ treatment, and (v) reversed osmosis treatment.

### 2.2. Batch Experiments

Aerobic and anaerobic batch experiments (microcosms) have been performed in duplicate in 120 mL bottles with medium and activated sludge from two conventional municipal treatment plants in The Netherlands: (1) adapted sludge from a sewage treatment plant and (2) unadapted sludge from a recently started membrane bioreactor. 

For the aerobic batch experiments, 120 mL bottles were filled with 40 mL aerobic medium and 2 mL sludge and sealed with butyl rubber or Viton rubber stoppers (Rubber BV, Hilversum, The Netherlands). The aerobic medium consisted of (per liter of demineralized water) 3.5 g Na_2_HPO_4_·2H_2_O, 1 g KH_2_PO_4_, 0.01 g Fe-ammonium citrate, 1 g (NH_4_)_2_SO_4_·7H_2_O, 0.04 g MgSO_4_·7H_2_O, and 8 mg CaCl_2_·2H_2_O. Vitamins and tracers were added according to the medium used by de Bruin et al. [21]. Autoclaved controls were taken along as well. 

For the batch experiments under anoxic conditions, the medium as described for the aerobic batches was prepared anaerobically while continuously flushing with N_2_ gas and the addition of nitrate (0.85 to 3.4 g/L NaNO_3_). Resazurin, a colour indicator to control anoxic conditions, was added at a final concentration of 0.5 *μ*g/L. After closing the bottles, the gas phase in the bottles was changed to 80% N_2_/20% CO_2_ (v/v) and brought to 1.3 bar. 

The pharmaceuticals ibuprofen and diclofenac were added at a concentration of 50 mg/L and in further experiments gradually increased to 250 and 300 mg/L, respectively. Initially, pharmaceuticals, dissolved in methanol, were added to the batches, after which the methanol was evaporated. This was only done in the first experiments with unadapted sludge. In later experiments, the pharmaceuticals were dissolved in small volumes of medium, filter sterilised, and added after autoclaving. 

Stimulated or cometabolic degradation of the pharmaceuticals was tested by the addition of acetate (5 to 50 mM) or toluene (12.5 to 250 *μ*M). Viton rubber stoppers were used when toluene was added to the batches. Acetate or toluene was measured routinely and readded upon depletion.

The bottles were incubated on a shaker (100 rpm) in the dark at 30°C. Autoclaved controls were taken along as well. The concentrations of ibuprofen or diclofenac were measured routinely. Oxygen or nitrate was measured routinely and added when depleted.

### 2.3. Enrichment of Bacteria

Batch cultures were used to further isolate the pharmaceutical degrading bacteria by dilution series in either aerobic liquid medium or on agar plates. Dilution series in medium were made in 115 mL serum bottles with 40 mL aerobic medium as described above, by transferring 4 mL from the batch culture to fresh medium, followed by transferring 4 mL of the latter into 40 mL fresh medium and repeated several times. The bottles were incubated on a shaker (100 rpm) in the dark at 30°C.

The agar plates were made with aerobic medium as described above and 15 g/L agar. After cooling of the medium, 0.1 mL of the microbial dilution series was spread on the agar plate with a sterile glass rod. The agar plates were placed upside down to prevent condensation on the agar and incubated in the dark at 30°C.

### 2.4. Molecular Analyses

The purity of the cultures was tested by Polymerase Chain Reaction (PCR) and Denaturing Gradient Gel Electrophoresis (DGGE). DNA was extracted using a FastDNA SPIN Kit for Soil (MP Biomedicals). Bacterial primers 968F GC, 1401R [22] and archaeal primers Arc344F GC [23] and Arc915R [24] were used for the amplification of the 16S rRNA. The final volume of 50 *μ*L PCR mixture contains 10 *μ*L 5x green gotaq reaction buffer (Promega), 1 *μ*L of each primer, 1 *μ*L deoxynucleoside triphosphate (dNTP), 0.25 *μ*L Taq DNA polymerase (Promega), and 5 *μ*L template DNA (sample). PCR was performed under the following conditions: preheating to 94°C for 2 min. or 5 min. for bacterial or archaeal PCR, respectively. For PCR using bacterial primers 35 amplification cycles of denaturation at 94°C for 30 sec., primer annealing for 40 sec. at 56°C, and extension for 1 min. at 72°C were performed followed by a final extension of 5 min. at 72°C. PCR using archaeal primers was performed using 35 cycles of denaturation for 10 sec. at 94°C, primer annealing for 20 sec. at 61°C, which was decreased every cycle until 56°C, and extension for 40 sec. at 72°C. This was followed by a final extension at 72°C for 30 min. 

DGGE analysis was performed as described before [25], and gels were stained with AgNO_3_ according to Sanguinetti et al. [26].

### 2.5. Analytical Procedures

Toluene was analysed on a Gas Chromatograph (GC; Shimadzu GC 2010) equipped with a Sil5 CB column (25 m; Chrompack, Middelburg, The Netherlands). Headspace samples (0.4 mL) were taken from the batch experiments and directly analysed for their toluene concentration on the GC. The GC was operated in constant flow mode, and the column temperature was 80°C. Toluene was detected with a Flame Ionisation Detector (FID) at 300°C. External standards of toluene at five different concentrations from 0 to 300 *μ*M were used for calibration.

The concentration of acetate was measured using High Performance Liquid Chromatograph (HPLC Varian, Middelburg, The Netherlands). Prior to analyses, liquid samples from the batch experiments were acidified with 0.04 M H_2_SO_4_ to pH 2, and 30 *μ*L sample was injected onto the column (Metacarb 67H 300 mm column; Varian, Middelburg, The Netherlands). The flow rate was 0.8 mL/min with an isocratic eluent of 0.01 N H_2_SO_4_ in Milli-Q water. Detection was performed using a UV detector at a wavelength of 220 nm. Sodium crotonate was used as an internal standard at a concentration of 6 mM, and external standards of sodium acetate at five different concentrations from 0 to 20 mM were used for calibration.

Oxygen was analysed by gas chromatography. Headspace samples of 0.4 mL were taken from the batch experiments and directly injected onto a Shimadzu GC-14B (Shimadzu, Kyoto, Japan) equipped with a packed column (Molsieve 13X, 60–80 mesh, 2 m length, 3 mm internal diameter; Varian, Middelburg, The Netherlands) and a thermal conductivity detector set at 70 mA. The GC was operated in constant flow mode, with an injector temperature of 80°C. The column temperature was 100°C, and the detector temperature was 130°C. Argon was used as the carrier gas at a flow rate of 30 mL/min. Calibration was done with air samples (21% oxygen) and 100% nitrogen samples.

Nitrate was analysed by anion exchange chromatography (Dionex DX-120l; Dionex Breda, The Netherlands). Samples (20 *μ*L) were injected onto an IONPAC AS22 column (Dionex, Breda, The Netherlands) operated at 35°C. The flow rate was set to 1.2 mL/min with an eluent of 4.5 mM Na_2_CO_3_ and 1.4 mM Na_2_CO_3_ in Milli-Q water. Bromide (1 mM) was added as an internal standard, and external standards of NaNO_3_ at five different concentrations from 0 to 20 mM were used for calibration.

Ibuprofen and diclofenac were analysed using liquid chromatography mass spectrometry (UPLC-MS/MS). Sample clean-up for the samples from the batch experiments consisted of centrifuging (5 min, 13,000 rpm) to remove the sludge, followed by diluting the supernatant with acetonitrile-water (40% : 60%) to obtain a maximum concentration of 5 mg/L. The injection volume was 20 *μ*L. Liquid chromatography consists of a Waters Chromatography Acquity UPLC separation module, equipped with an Acquity UPLC HSS T3 column (1.7 *μ*m ∗ 100 ∗ 2.1 mm ID) at 65°C. The LC mobile phase consisted of a mixture of 0.1% acetic acid in water (solution A) and 0.1% acetic acid in acetonitrile (solution B). A linear gradient was used with 60% B for 1 min, followed by an increase to 95% in 0.1 min, 3 min. at 95%, and finally a decrease to 60% in 0.1 min. 

Mass-spectrometer (MS) analysis was carried out with a Waters-Micromass Ultima Platinum. Depending on compound, the measurement was carried out in positive or negative electrospray ionisation (ESI) mode. The measurements were performed in negative ionisation mode with the following settings: a capillary voltage of 1.2 kV, a cone voltage of 35 V, RF lens 1 of: 5, aperture at 0.5, and RF lens 2 at 1.0. The source temperature was 120°C, and desolvation temperature was 325°C. The cone gas flow was 116 L/hr, and the desolvation gas flow was 701 L/hr. LM1/HM1 resolution was 14, with an ion energy of 0. LM2/HM2 resolution was 14.5, with an ion energy of 1.0. The collision cell pressure was 3.06e-03, and the collision cell entrance was 10, with a CE gain of 1 and exit 0. The MRM transitions for ibuprofen and diclofenac were, respectively, 205.0 > 161.1 and 294.0 > 250.0.

Internal standards of deuterated ibuprofen and diclofenac were used in concentrations of 1 mg/L. External standards of ibuprofen and diclofenac at five different concentrations ranging from 0 to 5 mg/L were used for calibration.

Intermediates were analysed by liquid chromatography time of flight mass spectrometry (LC-ToF-MS). Samples from the incubations were centrifuged (5 min, 13,000 rpm) to remove the sludge before injection onto the LC system, and 90 *μ*L was mixed with 10 *μ*L acetonitrile and acidified with acetic acid (final concentration 0.01%). The samples were injected at a Bruker microToF-Q coupled via an ESI interface to a Waters Acquity system with column oven. A Waters BEH-C18 column (150 mm × 2.1 mm, 1.7 *μ*m; 4.6 × 150 mm, df = 3.5 *μ*m) was used with a mobile phase A (water) and B (acetonitrile). Initially, 5% B was used for 0.3 min, followed by an increase of 80% in 5 min, an increase of 95% B in 0.1 min., maintained at 1 min., and finally an increase to its initial conditions of 5% B. The column oven was set to 60°C and the mobile phase flow was set at 0.6 mL/min. The injection volume was 50 *μ*L.

The LC-ToF-MS measurements were performed in negative ionization mode. Data were collected from 50 to 1000 *m/z* with a scan time of 1 second. The electrospray voltage of the ion source was set at 2500 V. The nebulizer gas flow was 2.0 bar, and drying gas flow was 5 L/min. The drying temperature was set at 200°C. The transfer time of the source was 100 *μ*s, and the hexapole radiofrequency (RF) was 400 Vpp.

The instrument was tuned and calibrated before analysis. At the beginning, during, and at the end of the analysis, an internal mass calibration solution was added via an injection port. This calibration solution contained sodium formate. By the use of this internal calibration mix the each individual compound was mass corrected. Instrument resolution (full width at half maximum) was approximately 20000 for *m/z* 10000. 

## 3. Results and Discussion

**Figure 2:**
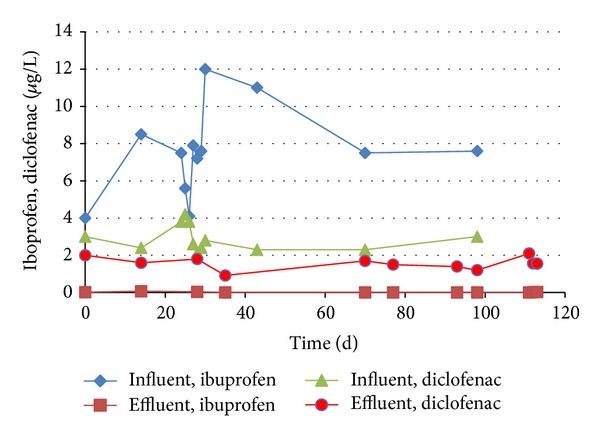
Influent and effluent concentrations of ibuprofen and diclofenac in a membrane bioreactor, operated with wastewater from a hospital.

**Figure 3:**
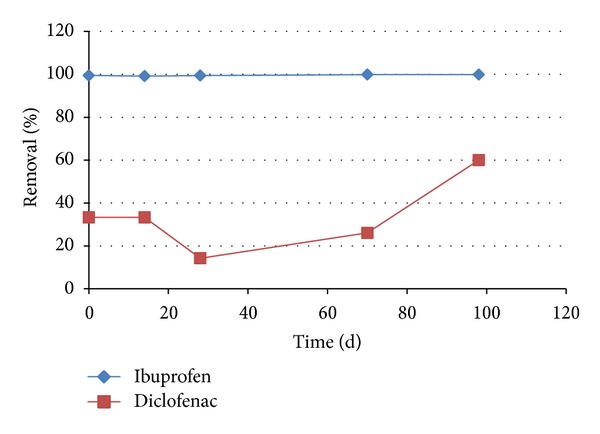
Removal percentages of ibuprofen and diclofenac in a membrane bioreactor, operated with wastewater from a hospital.

A pilot wastewater treatment plant treating hospital wastewater with a membrane bioreactor (MBR) and several posttreatment steps was evaluated for its removal of 44 pharmaceuticals, including ibuprofen and diclofenac. The MBR received concentrations that varied from 4 to 12 *μ*g/L ibuprofen and 2 to 4 *μ*g/L diclofenac (Figure 2). The concentration profile in the effluent was measured using UPLC-MS/MS during the first months after start-up and showed effective removal of ibuprofen below the detection limit of 0.01 *μ*g/L but not for diclofenac, yielding a residual diclofenac concentration (Figures 2 and 3). This poor biological degradation of diclofenac in the MBR can be due to various factors; for example, the degrading bacteria are not present, the degradation rate is too low (e.g., a higher hydraulic retention time is needed), environmental conditions are not suitable for degradation (nutrients, pH, temperature), and so forth. A higher retention time has previously been suggested for other treatment systems, where the influence of various HRT on the removal rate of pharmaceuticals was demonstrated [27].

Alternatively, physical-chemical posttreatment steps were needed to effectively remove pharmaceuticals from the wastewater. Various posttreatment steps were tested in this study for the removal of diclofenac; see Table 1. Ozonation and reversed osmosis were most effective in the removal of diclofenac, but also granular activated carbon showed a good removal of the present diclofenac (95%). Ibuprofen was not studied in the posttreatment steps, as it was already removed in our MBR.

To date, information on the treatment of pharmaceuticals in various treatment systems is scarce. The use of physical-chemical posttreatment steps has been described in a few studies and our results are in line with recent studies, where the use of activated carbon to adsorb pharmaceuticals has been successfully described [28–31]. The advantage of activated carbon is that it removes pharmaceuticals without the generation of toxic active products as by, for example, UV/peroxide oxidation or ozone treatment. Like our posttreatment results showed, these technologies have also been shown to effectively remove pharmaceuticals [7–10]. However, this may also lead to the formation of potentially harmful by-products, and such oxidation technologies are characterized by a large ecological footprint, due to a high energy use [4], which might make them less attractive for application.

To get more insight in the degradation processes in this MBR, the responsible microbes, and the underlying degradation mechanisms, laboratory experiments were performed to test the potential for aerobic and anaerobic degradation of ibuprofen and diclofenac. Microcosms were started with sludge from two treatment plants as inoculum; (i) a sludge that was adapted to concentrations of pharmaceuticals and (ii) unadapted sludge from an MBR reactor sampled shortly after start-up and only exposed to low concentrations of pharmaceuticals. Initial experiments were performed with both sludges, the individual pharmaceuticals in the presence of oxygen or nitrate, and the presence of an extra carbon and energy source, respectively, acetate or toluene. The bottles were fully closed to prevent any evaporation out of the bottle. Oxygen was measured routinely and added when depleted. 

**Figure 4:**
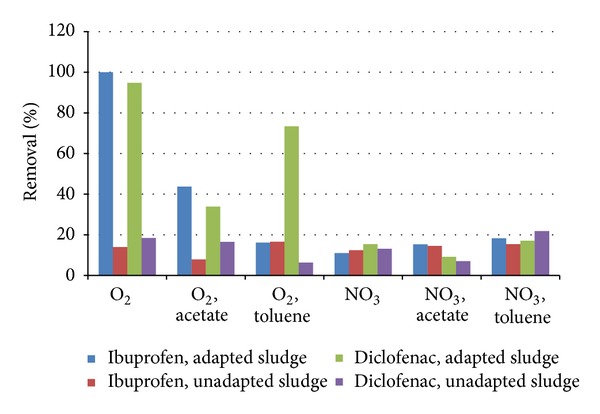
Degradation of 50 mg/L ibuprofen and diclofenac in batches with activated sludge from a municipal wastewater treatment plant and unadapted sludge with oxygen or nitrate after 27 days of incubation. Standard deviations in data shown were 10%.

Both ibuprofen and diclofenac were degraded in the presence of oxygen within 27 days of incubation (Figure 4). Controls without sludge or without electron acceptors (oxygen and nitrate) did not show any decrease in the concentration of the two pharmaceuticals (results not shown). The aerobic degradation of both ibuprofen and diclofenac in the incubations with adapted sludge was two to five times faster compared to the incubations with the sludge from the MBR (Figure 4). This indicates that the microbial populations in the two inocula show a different affinity towards the degradation of the selected pharmaceuticals. For diclofenac, this effect was more pronounced in the MBR, with no diclofenac removal (Figure 3), compared to the initial microcosms that showed a slow degradation. Theoretically, the reduced degradation in the batches with MBR inoculum may also be affected by the presence of methanol in the initial batches, that was used to dissolve the pharmaceuticals to the batches. However, the lower removal rate in the MBR itself is related to the microbial population present. Other studies describe diverse effects of the presence of biodegradation activity prior to elevated exposure. After providing higher concentrations, either comparable or higher biological degradation rates were found [32].

Acetate or toluene was added to the batches to promote biomass growth, suggesting that they could serve as a primary substrate in a cometabolic transformation of the pharmaceuticals. In our experiments, the addition of acetate or toluene in the initial incubations resulted in a slower degradation of ibuprofen and diclofenac, most likely due to competition for the different substrates and electron acceptors, similar to competing substrate consumption in raw or treated wastewater [17]. Other studies that tested the effect of acetate dependent behaviour of ibuprofen and diclofenac degradation showed varying results ranging from no effect of acetate, to acetate concentration controlled degradation [33, 34]. This shows that the concentration and character of other organic carbon present in wastewater effluents can affect the degradation efficiency of pharmaceuticals. The differences in the trends of these literature data and our studies are likely due to different experimental designs (microcosms, column studies). These studies vary in retention times, temperature, the presence of nutrients, and so forth, and highlight the need for further research to study parameters that affect the transformation of pharmaceuticals.

**Figure 5:**
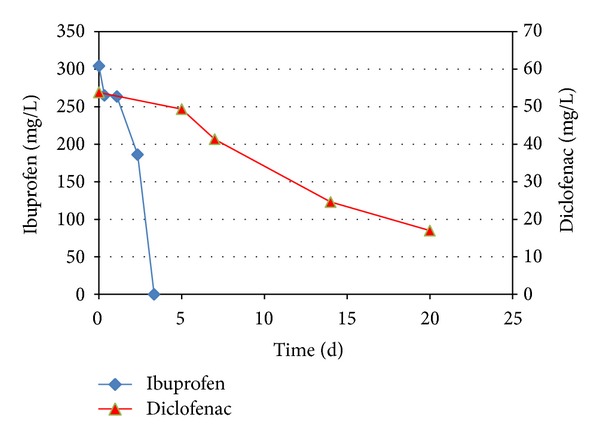
Degradation of ibuprofen and diclofenac in microcosms, after 2 subsequent transfers, resulting in 2nd generation cultures. Standard deviations in data shown were 4%.

After the degradation of the initial concentration of 50 mg/L in the incubations with ibuprofen as carbon and energy source, the concentration ibuprofen was gradually increased to 75 mg/L, 100 mg/L, and a final concentration of 250 mg/L. This highest concentration of ibuprofen (250 mg/L) was degraded within 4 days (Figure 5). The diclofenac concentration was not increased but kept at 50 mg/L since the degradation rate was much lower. 

All microcosms under anoxic conditions with nitrate as electron acceptor showed no significant degradation of the pharmaceuticals (Figure 4), and these conditions were not further studied in this research. Our experiments were performed with aerobic sludge as inoculum, providing predominantly a pool of aerobic bacteria that were not adapted to anoxic conditions. As many nitrate reducing bacteria are also able to reduce nitrate, our inoculum should be suitable to enrich bacteria that could degrade diclofenac with nitrate as electron acceptor. Nevertheless, we were not able to establish degradation with nitrate as electron acceptor. Other research has reported an initial decrease in diclofenac concentration in batch experiments with nitrate as electron acceptor, but considering the concentration established towards the end of the experiments, they concluded that no overall removal of diclofenac occurred [35]. Whether sorption or desorption played a role in the rebounding of the concentration was not elucidated by the authors.

The degradation of ibuprofen was fast (50 mg/L within 27 days) in the aerobic microcosms, and no intermediates could be detected by LC-ToF-MS analyses during the degradation. Known intermediates that have been detected in incubations with activated sludge are hydroxyibuprofen, carboxyibuprofen, and carboxy hydratropic acid [36]. Isobutylcatechol, 5-formyl-2-hydroxy-7-methylocta-2,4-dienoic acid and 2-hydroxy-5-isobutylhexa-2,4-dienedioic acid were detected during the degradation of ibuprofen with *Sphingomonas* sp. strain Ibu-2 [20]. Recently, also 1,2-dihydroxy ibuprofen was identified as an intermediate [37]. None of those could be detected in our degradation studies, indicating either a different degradation pathway, or more likely, a mineralization to CO_2_ and H_2_O without the accumulation of intermediates.

When diclofenac was degraded, eight different metabolites were observed in our enrichments, of which only three have been found in other studies: 2-((2,6-dichloro-phenyl) amino)benzyl alcohol methyl ether [38], and the undefined compounds M190 and M340 with a mono-isotopic mass of 322.99673 and 338.9953, respectively, as described by Pérez and Barceló [39]. LC-ToF-MS analyses showed five further intermediates with a mono-isotopic mass of, respectively, 305.9835, 310.9984, 315.9923, 325.9869, and 383.9797. Identification of these metabolites was not achieved, making it impossible to further identify the degradation mechanisms of ibuprofen or diclofenac in our study. In general, hydroxylated metabolites are most likely to be formed during the aerobic degradation of diclofenac although we did not find such compounds in our batch experiments.

After several transfers into fresh media, aerobic enrichment cultures with ibuprofen or diclofenac as carbon and energy source degraded the pharmaceuticals up to concentrations of 250 mg/L ibuprofen or 300 mg/L diclofenac. Higher concentrations were not tested. These concentrations (mg/L) are significantly higher than those found in wastewater treatment plants (*μ*g/L instead of ng/L) but were needed for our experimental set-up to transfer, enrich, and obtain purified bacterial cultures.

**Figure 6:**
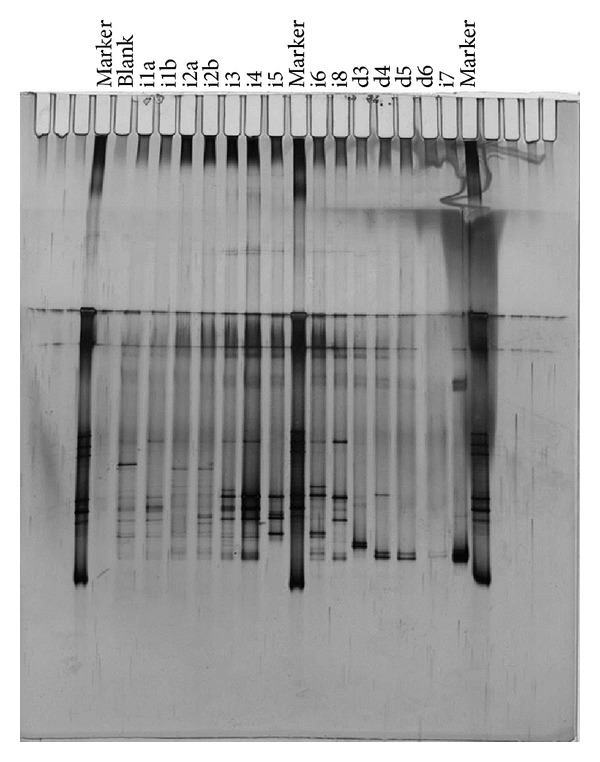
DGGE gel loaded with PCR products from enrichment cultures and purification experiments. The sample ID is given on top of the gel, and is from left to right: marker, blank, i1a, i1b, i2a, i2b, i3, i4, i5, marker, i6, i8, d3, d4, d5, d6, i7, marker.

These batches were used to further isolate the pharmaceutical degrading bacteria by dilution series in either liquid medium or on agar plates and were used to obtain bacterial cultures on ibuprofen and diclofenac. Single colonies on agar plates were inoculated in fresh liquid media and showed prolonged degradation of the pharmaceuticals. In order to link observed degradation with the purity of the microbial community during the degradation, an assessment of the microbial population was made through DGGE profiling. Molecular analyses of ibuprofen and diclofenac degrading cultures showed further enrichment along the dilution series when looking at the DGGE profile of the various samples (Figure 6). The highly enriched cultures on ibuprofen (i7) or diclofenac (d6) both originate from an isolated culture on an agar plate, and show a DGGE profile with only 2 bands. This indicates the presence of a highly enriched bacterial culture with only one species with two copies of the 16s DNA or two different bacterial species. This demonstrates that specific ibuprofen or diclofenac degrading bacteria were present in the original used inoculum from the wastewater treatment plants, and that they can be used to enhance the treatment of the pharmaceuticals in the wastewater. The necessity of a complete removal of pharmaceuticals from wastewater will stimulate water reuse technologies, addressing the worldwide increasing demand for clean fresh water resources. For this, the development of innovative, effective, and sustainable microbiological removal technologies for organic micropollutants in waste water is needed. 

## 4. Conclusions

Our study shows that the pilot MBR treating hospital waste removed ibuprofen below the detection limit of 0.01 *μ*g/L whereas diclofenac was not removed in the MBR. Posttreatment was needed for an efficient removal of all pharmaceuticals in the wastewater.

The pharmaceuticals were aerobically degraded in microcosms, with ibuprofen being faster degraded than diclofenac. The degradation was not enhanced by the presence of acetate or toluene as primary substrates, and the degradation of the pharmaceuticals in the presence of nitrate as electron acceptor was not observed. 250 mg/L ibuprofen was degraded within 4 days to undetectable concentrations, and 75% of the added 300 mg/L diclofenac was degraded within 3 weeks. Higher concentrations were not tested.

Our experiments showed the degradation of both ibuprofen and diclofenac, with no intermediates detection for the degradation of ibuprofen, whereas several intermediates of the degradation pathway of diclofenac were identified. DGGE analyses of the enrichment culture on ibuprofen or diclofenac showed that highly purified cultures were obtained, with only two bands on the DGGE gel, indicating either one or two bacterial species.

## Figures and Tables

**Table 1:** Removal percentage of diclofenac in various posttreatment systems of the membrane bioreactor.

Posttreatment	Diclofenac removal
Granular activated carbon (GAC)	95.0%
Ozone unit	99.5%
Ozone unit with H_2_O_2_ addition	99.5%
Ozone unit and GAC	99.5%
UV/H_2_O_2_-system	80.0%
Reversed osmosis	99.5%
